# Piezo channels in physical field therapy: From mechanotransduction mechanisms to bioelectromagnetic modulation and biomedical applications

**DOI:** 10.1016/j.mbm.2026.100207

**Published:** 2026-07-23

**Authors:** Bing Li, Hui-Ming Kang, Wei-Dong Pan, Ye Li

**Affiliations:** a Beijing Key Laboratory of Bioelectromagnetics, Institute of Electrical Engineering, Chinese Academy of Sciences, Beijing, 100190, China; b School of Electronic, Electrical and Communication Engineering, University of Chinese Academy of Sciences, Beijing, 101408, China; c Department of Rehabilitation Sciences, The Hong Kong Polytechnic University, Hong Kong Special Administrative Region, 999077, China

**Keywords:** Piezo channels, Mechanotransduction, Physical field therapy, Bioelectromagnetic intervention, Piezoelectric biomaterials

## Abstract

The Piezo family of mechanosensitive ion channels serves as the primary molecular transducer that converts mechanical stimuli into electrochemical signals in living organisms, marking a pivotal breakthrough in mechanobiology. This review systematically summarizes recent advances in Piezo channel research, with a specific focus on the Piezo1, from the novel perspective of physical field therapy. We first describe the unique trimeric “propeller–nano-bowl” architecture of Piezo channels and their gating mechanisms, which rely on membrane tension sensing and a lever-like mechanical amplification principle. Next, we comprehensively explore the physiological roles of Piezo-mediated signaling in core systems, including the skeletal, cardiovascular, nervous, and immune systems, and elucidate their pathophysiological implications in diseases such as genetic disorders, osteoarthritis, and cancer. Finally, we highlight cutting-edge bioelectromagnetic intervention strategies, including the use of ultrasound and magnetic fields for non-invasive modulation of Piezo activity, as well as the application of piezoelectric biomaterials designed to mimic Piezo functions for tissue regeneration, neuromodulation, and targeted drug delivery.

## Highlights

• Piezo channels feature a unique “propeller–nano-bowl” architecture and a lever-like gating mechanism.
• They play essential roles in the skeletal, cardiovascular, neural, and immune systems.
• Piezo dysfunction is linked to genetic disorders, osteoarthritis, and cancer.
• Ultrasound and magnetic fields enable non-invasive modulation of Piezo activity.

## Graphical abstract

**Figure:**
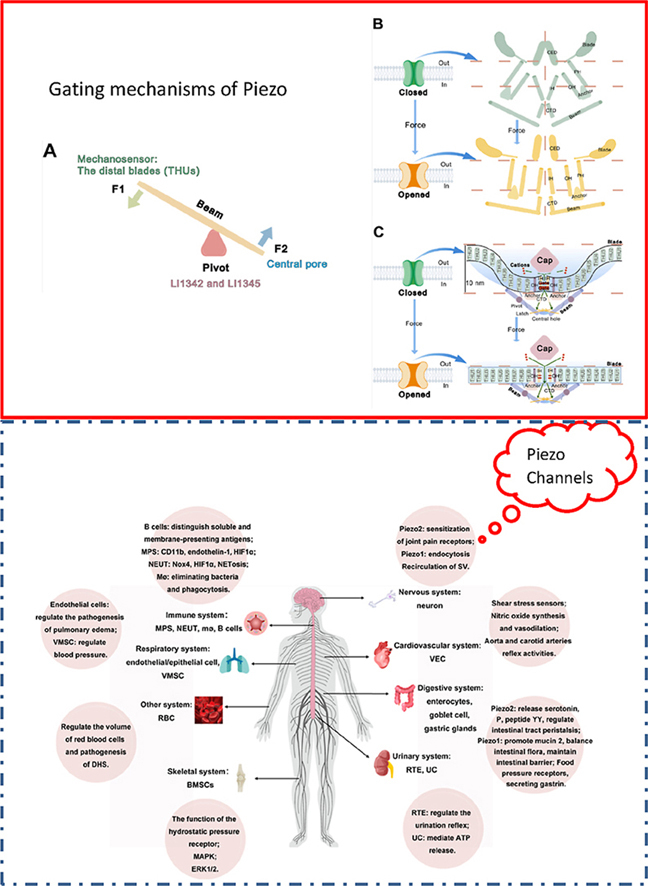

## 1 Introduction

Mechanotransduction refers to the process by which cells convert mechanical forces into biological activities. Mechanical forces are fundamental determinants of morphogenesis and cellular activities of all living organisms.^1^ Mechanotransduction converts mechanical stimuli into biochemical signals or bioelectric signals, thereby activating signaling pathways and influencing the functions of cells.^2^ The mechanotransduction process mainly relies on mechanosensitive ion channels, namely mechanosensors, to trigger calcium signals in non-excitable cells or depolarize the cell membrane in excitable cells, thereby activating a series of downstream signaling pathways and ultimately regulating specific cells or physiological metabolic activities 3, 4, 5. Mechanosensitive ion channels are transmembrane proteins that act as mechanosensors to convert mechanical stimuli into biological electrical signals. Mechanosensitive ion channels regulate many important physiological metabolic activities, such as bone homeostasis, pain, blood pressure, and proprioception, through the control of ion current generation by mechanical forces 6, 7, 8. Some researchers have reported that mechanosensitive ion channels include: the transient receptor potential (TRP) superfamily, two-pore domain potassium (K2P) channels, big potassium channel (BK), voltage-gated Ca^2+^ channels, Piezo channels, the epithelial sodium channel/degenerin (ENaC/DEG) family, and transmembrane channel-like 1/2 (TMC1/2). These mechanosensitive ion channels can also be activated by thermal or chemical stimuli, and they are closely related to various diseases and pathological conditions, such as pulmonary arterial hypertension, cancer, and osteoarthritis, etc. 8, 9, 10, 11, 12, 13, 14, 15.

Among these ion channels, the Piezo channel family was discovered to comprise two homologous members, the Piezo1 and Piezo2. In recent years, the Piezo channels have been increasingly recognized as broadly distributed mechanosensors involved in multiple physiological systems. In the skeletal system, the Piezo1 acts as a hydrostatic pressure sensor in bone marrow mesenchymal stem cells and couples mechanical loading to p38 MAPK/ERK1/2 activation, BMP2 expression, Runx2/Osterix generation, and osteogenic differentiation 16, 17, 18, 19, 20. In the cardiovascular system, Piezo1 senses blood-flow-induced mechanical deformation and shear stress in endothelial cells, converts mechanical signals into electrical signals, maintains endothelial homeostasis, and participates in nitric-oxide-mediated vasodilation and blood pressure control 21, 22, 23. In the nervous system, the Piezo2 functions as a major mechanotransduction ion channel for human proprioception, and the Piezo2 dysfunction is associated with sensory ataxia, distal arthrogryposis, Angelman syndrome, osteoarthritis-related mechanical pain, and other pain-related disorders 24, 25, 26, 27, 28, 29. In epithelial organs, the Piezo channels participate in gastrointestinal mechanosensation and hormone/neurotransmitter release, urinary stretch sensing and ATP-mediated urination reflexes, and respiratory mechanoregulation involving pulmonary vascular function and breathing reflexes 30, 31, 32, 33, 34, 35, 36, 37, 38, 39, 40, 41, 42, 43, 44, 45, 46, 47, 48, 49, 50, 51. In pathological microenvironments, the Piezo channels are involved in immune-cell activation, inflammatory responses, pulmonary hypertension, pulmonary edema, non-small cell lung cancer, and red-blood-cell volume disorders ^47^^,^^48^^,^^52^, ^53^, ^54^, ^55^, ^56^, ^57^, ^58^, ^59^, ^60^, ^61^, ^62^, ^63^, ^64^. While the Piezo1 functions as a ubiquitous mechanotransducer with high expression in the bladder and lungs, the Piezo2 is predominantly expressed in sensory neurons, mediating proprioception, touch, and nociception. Emerging evidence indicates that the Piezo channels are intricately involved in diverse pathological processes, ranging from cell proliferation and innate immunity to volume regulation. Consequently, the Piezo channel dysfunction has been implicated in a myriad of human diseases, including hereditary xerocytosis, lymphatic dysplasia, and various cancers ^15^^,^^52^^,^^53^^,^^65^, ^66^, ^67^, ^68^, ^69^, ^70^, ^71^, ^72^.

In recent years, studies have shown that the structure of Piezo is a three-leaf propeller-like homotrimer containing 38 transmembrane regions, with characteristic blade, cap, and beam structures.^15^^,^^72^ The Piezo1 and Piezo2 share similar structural features.^15^^,^^72^ A lever-like mechanotransduction mechanism and a curvature-based membrane tension sensing mechanism have been proposed to explain the gating of Piezo channels ^15^^,^^72^, ^73^, ^74^, ^75^. The upper and lower transmembrane protein gating activation mechanism of Piezo is controlled by the cap-like structure at the top of the protein, and the lateral plug gating is regulated by a plug-locking mechanism controlled by the leaves, and these two gating regulatory mechanisms may jointly regulate the mechanotransduction process of the Piezo protein 72, 73, 74, 75.

Piezo-mediated Ca^2+^ signaling is an important mechanism linking mechanical stimulation to downstream biological responses. In immune cells, the Piezo1 activation regulates intracellular and extracellular Ca^2+^ concentrations, induces Ca^2+^ influx in neutrophils and macrophages, and further promotes Nox4 and HIF1α expression, neutrophil extracellular trap formation, macrophage phagocytosis, and inflammatory responses ^52^^,^^55^, ^56^, ^57^, ^58^, ^59^, ^60^. In intestinal epithelial cells, the Piezo2 activation induces Ca^2+^ influx and increases intracellular Ca^2+^ concentration, thereby promoting mechanosensitive serotonin release, substance P and peptide YY secretion, and bioelectrochemical communication with enteric neurons that regulate intestinal peristalsis and secretion 35, 36, 37. In urinary epithelial cells, stretch-induced Piezo1 activation triggers Ca^2+^ influx and ATP release, while Piezo1/Piezo2-mediated ATP release contributes to urination reflex regulation 40, 41, 42, 43, 44. In red blood cells, gain-of-function mutations of Piezo1 enhance Ca^2+^ influx and electrochemical signaling under mechanical stimulation, contributing to abnormal red-blood-cell volume regulation.^54^^,^^63^^,^^64^ Together with Piezo1-mediated p38 MAPK/ERK1/2 signaling in osteogenic differentiation and nitric-oxide-mediated vascular relaxation, these findings indicate that Piezo channels connect mechanical cues with Ca^2+^-dependent, electrical, electrochemical, and molecular signaling events across different physiological systems 18, 19, 20, 21, 22, 23.

In addition to mechanical forces, other physical stimuli such as magnetic fields also play important roles in regulating biological activities. In the field of biology, there are numerous biological phenomena that respond effectively to magnetic fields. For instance, under magnetic field conditions, plants can regulate their calcium signal transduction and proline pathways, thereby enhancing their stress resistance and protecting themselves from both biotic and abiotic stresses.^76^ Static magnetic fields with specific parameters have been shown to alleviate pain in humans and mice.^77^^,^^78^ Static magnetic fields can promote human bone regeneration, wound healing, and regulate stem cells, etc..^78^^,^^79^ Magnetic fields can be classified into static magnetic fields, rotating magnetic fields, pulsed magnetic fields, and alternating magnetic fields based on their direction, source, and induction intensity. Among them, static magnetic fields are the most fundamental form and the basis of other types of electromagnetic fields.^80^ Currently, the types of electromagnetic fields mainly used in magnetic therapy are static magnetic fields and pulsed magnetic fields 80, 81, 82. A large number of magnetic biology studies have shown that static magnetic fields can affect the differentiation of bone marrow mesenchymal stem cells, thereby having a significant impact on bone-related diseases such as fractures and bone healing.^80^ Other studies have shown that pulsed magnetic fields can accelerate the healing of bone tissue by activating cellular metabolic activities, improve the quality of bone tissue repair, and have the effects of reducing pain and treating osteoarthritis and inflammatory diseases of the musculoskeletal system. They can also promote wound repair and healing by influencing blood microcirculation and improving the supply of nutrients to the injured area.^81^^,^^82^ In addition, extremely low-frequency electromagnetic fields have obvious improvement effects on the mental and functional states of stroke patients.^82^

The piezoelectric effect refers to the phenomenon where certain materials produce electrical polarization when subjected to mechanical pressure.^83^ With the advancement of research, scholars have discovered the piezoelectric effect in numerous biological systems, such as skin, bones, and tendons, and the bioelectricity generated by the piezoelectric effect in living organisms holds significant physiological importance 84, 85, 86, 87, 88. The Piezo channel is the main mechanosensors on the cell membrane of human body cells.^66^^,^^89^ Therefore, piezoelectric materials are used to solve the tissue repair problems in the biomedical field by mimicking the natural process of mechanical conversion by Piezo in the body.^90^

The innovative piezoelectric microneedles based on ultrasound can release local electric charges under ultrasound irradiation, providing local electrical signals. These signals are transmitted downward, thereby achieving the purpose of regulating specific metabolic activities in a specific area and within a specific time frame.^91^ Studies have also shown that ultrasound waves and the ultrasonic piezoelectric effect have good application prospects in the treatment of infectious bone defects.^92^ Ultrasound therapy can destroy the biofilm structure of bacteria through physical mechanisms and also promote the penetration of antibiotic drugs into target bacteria to break the limitations of traditional treatment.^93^ Moreover, when using ultrasound therapy, when ultrasound waves act on piezoelectric materials that can produce polarization phenomena under mechanical pressure, it can trigger the generation of an electric field, thereby promoting the proliferation and differentiation of osteoblasts and accelerating the bone injury repair process, achieving better therapeutic effects.^94^^,^^95^ It has been found that the combined treatment method of magnetic stimulation and electrical stimulation can indeed significantly promote cell proliferation and differentiation.^96^ Ultrasound microbubbles are composed of materials such as proteins and phospholipids encapsulating gas, and have good biocompatibility and are widely used in the biomedical field, such as for achieving targeted drug transportation.^92^ Under the action of ultrasound waves, ultrasound microbubbles can generate powerful microjets and shear forces, undergo cavitation effects, and release the encapsulated antibacterial drugs into the environment with target bacteria to achieve good sterilization effects.^97^^,^^98^

## 2 The structural basis and mechanical electrical transduction mechanism of Piezo channels

### 2.1 Structure of Piezo

#### 2.1.1 Piezo1

**Fig. 1:**
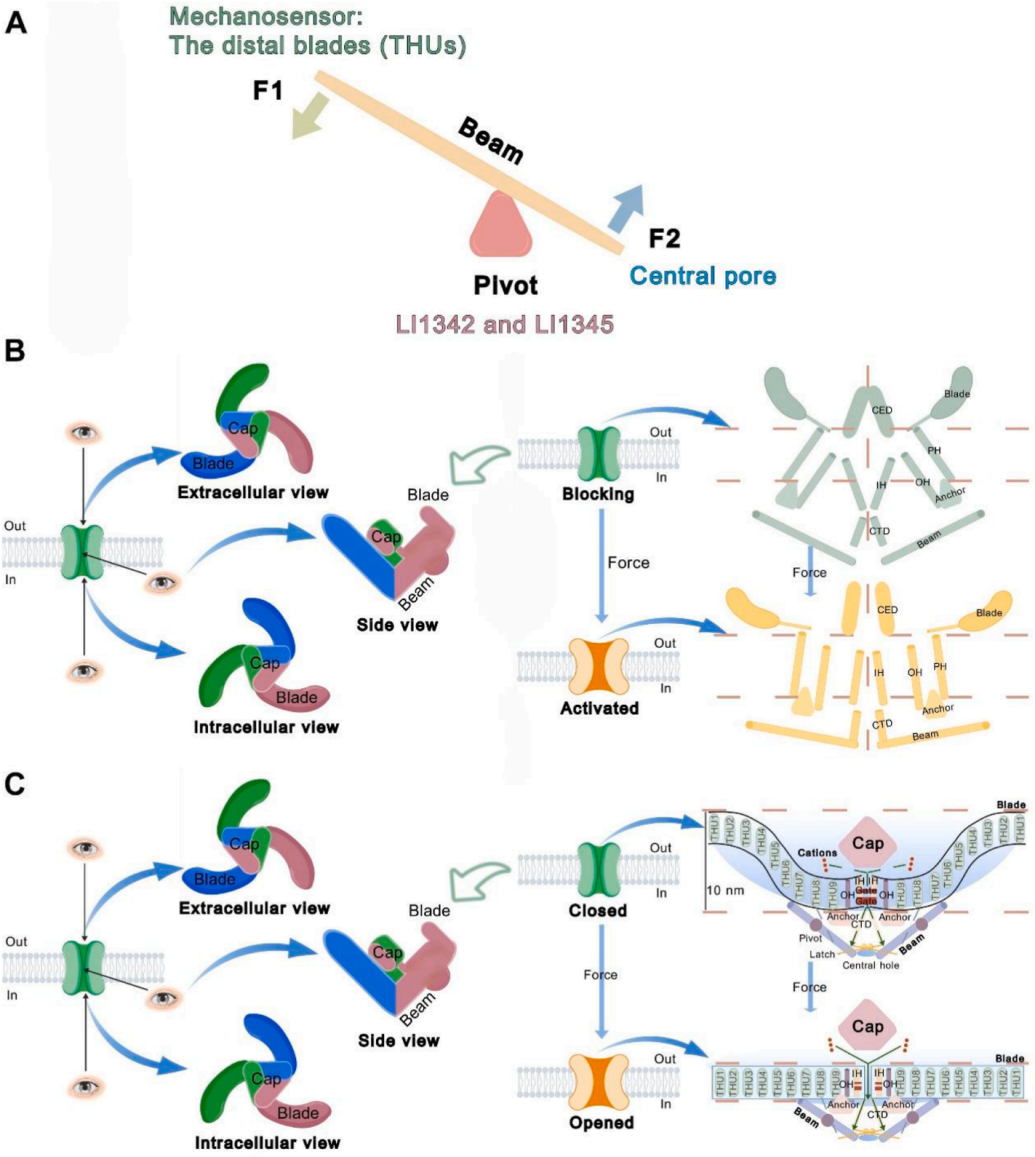
Structural view and two gating mechanisms of Piezo. A and B:The Lever-like mechanotransduction; C: The curvature-based gating mechanism.

The Piezo1 protein is composed of 2547 residues and is formed by a homologous trimer with a three-leafed propeller-like structure. It consists of: three outer propeller-like structures, a cap-like structure at the top, an ion-conducting channel in the center, three long beams located on the inner side connecting the three propeller-like structures and the cap-like structure, and a transmembrane region between these structural features as illustrated in the structural model (Fig. 1).^15^^,^^72^

Piezo1 has 38 transmembrane regions, and high-resolution structural analyses of each subunit of this protein have been conducted by scholars, revealing its unique 38-TM topology, which includes a C-terminal pore module, N-terminal blades, connecting long beams, and anchoring domains.^72^^,^^99^ The inner and outer helices are the two transmembrane regions TM37 and TM38 closest to the center of the protein, surrounding the central transmembrane pore. The remaining transmembrane regions TM1-TM36 form a curved blade-like structure composed of 9 repeated folds, each containing 4 transmembrane regions, and this fold is called a TM helical unit (THU).^15^^,^^72^ Additionally, the connecting long beam, which is 9 nm long, is located beneath the curved blade and connects the transmembrane helical units (THUs) and the central pore structure.^72^

The amino acid residues 2210-2457 of the Piezo1 protein form an extracellular loop, which is located after the last transmembrane region at the C-terminus and is called the C-terminal extracellular domain (CED).^100^ The amino acid residues 2218-2453 of the Piezo1 protein trimerize to form a central cap-like structure, and the C-terminal extracellular domain forms a trimeric complex to constitute the central cap-like structure, with an open extracellular vestibule (EV) in the middle of the central cap-like structure.^101^^,^^102^

The Piezo1 protein contains a hairpin structure composed of three helices, α1, α2, and α3, which connects the inner and outer helices surrounding the central transmembrane pore to the C-terminal extracellular domain (CED) plane. This structure is called the “anchor”, and the α1 and α2 helices in this structure form an inverted V shape to ensure the integrity of the ion conduction channel.^15^ Proteins interacting with Piezo, such as SERCA2, inhibit the Piezo protein by acting on the linker between the anchor and the outer helix. Studies have found that mutations in the linker between the anchor and the outer helix can lead to severe diseases.^54^^,^^99^^,^^103^ Therefore, the structure and function of the anchor region are of great importance.^15^

The Piezo1 protein has three long beam structures, each 9 nm in length, composed of amino acid residues H1300-S1362. These long beam structures form a 30° angle with the plane of the cell membrane surface.^15^ These three long beam structures not only connect the distal transmembrane helical units (THUs) and the central ion conduction channel but also support the framework of the entire transmembrane region.^15^

The Piezo1 protein contains three highly curved blades, each of which is clockwise curved. Each blade is composed of 9 transmembrane helix units (THUs), and each blade forms a L-shaped helical structure, which is composed of five transmembrane structural regions: TM29, TM25, TM21, TM17, and TM13.^15^ The TM13-TM24 on the blade is located on the highly curved membrane surface and forms part of the nano-bowl. Therefore, this indicates that the Piezo1 protein can bend the membrane where it is located, that is, the Piezo1 is regulated by the curvature and tension of the cell membrane.^15^^,^^104^^,^^105^

The center of the Piezo1 protein contains an ion-conducting channel. This central pore is composed of amino acid residues from 2189 to 2547 and includes two transmembrane regions. The ion channel runs from the inner side of the cell to the outer side as follows: the intracellular vestibule (IV) below the cell membrane, the transmembrane vestibule (MV) within the cell membrane, and the extracellular vestibule (EV) located within the cap-like structure outside the cell membrane. Among them, the intracellular vestibule (IV) and the extracellular vestibule (EV) each have an opening that can be connected to the transmembrane vestibule (MV).^15^ The amino acid residue fragment of residues 2393-2397 located in the central cap (i.e., the C-terminal domain (CED)) plays an important role in ensuring efficient ion conduction and the preferential conductance of cations over anions.^15^ The two amino acid residues E2495 and E2496 located in the intracellular vestibule (IV) are crucial for blocking the ion conductive channel and for the selectivity of Ca^2+^.^15^

#### 2.1.2 Piezo2

The Piezo2 protein is composed of 2822 amino acid residues and has a sequence homology of 42% with the Piezo1 protein. The Piezo2 protein also contains 38 transmembrane structural regions, and previous studies have determined these 38 transmembrane structural regions of the Piezo2 protein 72,106. When observing the Piezo2 protein from the side, one can see that the cap-like structure and the paddle-like structure form a nano-bowl structure with a depth of 9 nm and an opening diameter of 24 nm as illustrated in the structural model (Fig. 1).^72^ The long beam structure of the Piezo2 protein and the charged residues between the C-terminal domain are crucial for the mechanical sensitivity of the channel.^107^ Compared to the structure of Piezo1 protein, the Piezo2 protein has additional contraction sites at amino acid residues E2757, F2754 and L2743.^106^ These additional contraction sites might be transmembrane gates controlled by the cap structure.^106^ Piezo2 and Piezo1 proteins have extremely similar structural features, with an overall structure similarity (the transmembrane structural regions of the three blades are highly curved), and both have 38 transmembrane structural regions and key domains, indicating that they may have a relatively similar mechanotransduction mechanism.^15^^,^^72^ The structure of the Piezo protein is shown in the following Fig. 1.

### 2.2 Mechanotransduction mechanism of Piezo channels

**Fig. 2:**
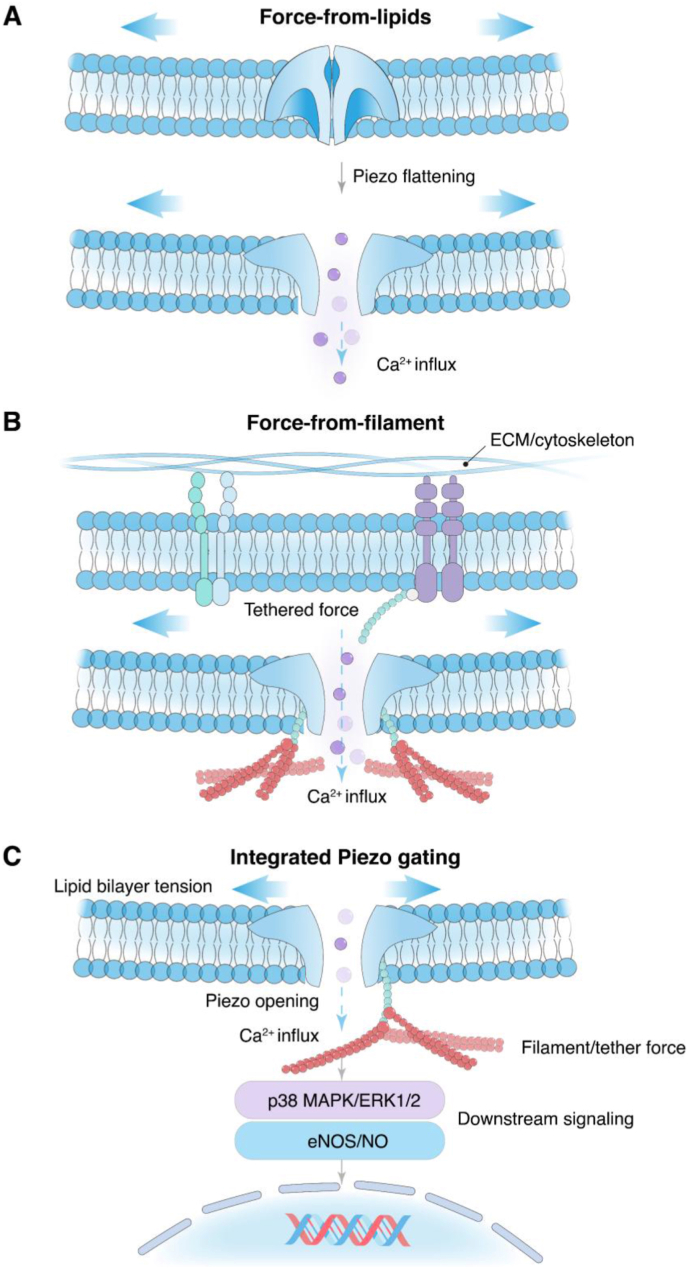
General model of mechanosensitive ion channels and schematic diagram of the piezo mechanotransduction process. A: General model of mechanosensitive ion channels - lipid force model; B: General model of mechanosensitive ion channels - filament force model; C: Piezo mechanotransduction process.

From the general gating principles of mechanosensitive ion channels, mechanotransduction by Piezo channels is not determined solely by a single structural change, but rather regulated collectively by multiple sources of mechanical energy. The two most representative mechanisms are the force-from-lipids model and the force-from-filament (force-from-tether) model.^108^^,^^109^ In the force-from-lipids model, mechanical energy is primarily transmitted directly to the channel protein through the lipid bilayer in the form of membrane tension, without relying on other cellular components such as the cytoskeleton or extracellular matrix. This mechanism has been shown to be sufficient to drive Piezo1 opening and forms a crucial basis for Piezo1's sensing of membrane tension and changes in membrane curvature, as shown in Fig. 2 A.^108^ In contrast, the force-from-filament (force-from-tether) model proposes that mechanical forces can be transmitted to mechanosensitive ion channels via the extracellular matrix, cell adhesion structures, cytoskeleton, or associated linker proteins, thereby directly or indirectly inducing conformational changes in the channel and modulating its opening, as shown in Fig. 2 B.^109^ It should be noted that these two models are not mutually exclusive, but instead work together within cell-specific mechanical microenvironments to distribute, amplify, and transmit forces. Therefore, the gating process of Piezo channels should be understood as an integrated mechanotransduction mechanism jointly regulated by lipid membrane tension, membrane curvature, cytoskeletal coupling, and extracellular matrix/adhesion structures, as shown in Fig. 2C.^108^^,^^109^

#### 2.2.1 Force-from-lipids model and curvature-based gating mechanism

The curvature-based gating mechanism of Piezo can be regarded as a representative form of the force-from-lipids model, because membrane tension and lipid-bilayer deformation directly regulate Piezo conformational changes and channel opening. By leveraging its inherent curvature, significant and reversible deformability, and structural rearrangement, the Piezo1 protein can sense changes in membrane curvature and tension, thereby exhibiting high mechanical sensitivity and selective ion permeation.^1^ Piezo1 protein exhibits varying degrees of mechanical sensitivity under different mechanical stimuli and in different environments. Early studies have found that Piezo1 is highly sensitive to lateral membrane tension, and mechanical stimulation of the lipid bilayer membrane can trigger Piezo channels. The resting tension of the lipid bilayer membrane will deactivate the Piezo1, and Piezo1 protein regulates its mechanical sensitivity in this way.^105^^,^^110^ Additionally, previous research has shown that Piezo proteins may conduct mechanotransduction through a dual gating mechanism. The transmembrane gating is regulated by the cap-like structure at the top, and the lateral plug gating is regulated by a locking mechanism controlled by the blade.^72^ A short peptide acts like a “plug”, blocking the central hole and each side hole of Piezo. The locking peptide located at the junction of the C-terminal domain and the extracellular domain at the C-terminus is also a short peptide. The three plugs on the side holes are fixed on the central axis of Piezo1, and are connected through hydrogen bonds with the locking peptide. Mechanical stimulation may act on the blade, and then the Piezo activation signal can be transmitted to the central axis to open the ion channel.^72^^,^^111^^,^^112^ Some scholars have conducted research and found that when force is applied in the direction perpendicular to the lipid bilayer membrane, the Piezo1 protein undergoes a significant flattening deformation. The area of this flattened structure is approximately 300 nm^2^, and this deformation is reversible.^113^^,^^114^ The huge and curved transmembrane regions of Piezo form a unique “nanobowl” or “dome” structure. The membrane tension causes the Piezo1 protein to deform from the “nanobowl"-like curved conformation to a flat state as illustrated in Fig. 1C.^113^^,^^114^ The blades in Piezo1 protein will flatten, the cap-like structure will detach and rotate, and the long beam structure will bend. These phenomena cause the ion conduction channel to open.^113^^,^^114^ Furthermore, existing studies have provided evidence that the flat and curved structures of Piezo1 are consistent with the shapes of water droplet and D-shaped lipid vesicles. This indicates that the Piezo1 protein can bend the lipid bilayer membrane and respond to changes in membrane tension related to the curvature of the membrane.^114^ After the Piezo1 protein undergoes a flat structure change in response to membrane tension, the expanded membrane plane area of 300 nm^2^ is consistent with the changes in membrane tension and the free energy required for the ion-conducting channel to shift from closed to open state.^114^ Additionally, through research, it was observed that while the Piezo protein undergoes a flattened structure deformation, the transmembrane gate and lateral plug gate also change.^114^ These research results can all support the hypothesis of curvature-based membrane tension gating mechanism. However, this model still requires determining the curvature of Piezo1 in its resting state to characterize it.^1^ Since the structures of Piezo1 and Piezo2 proteins are very similar, both having curved and closed structures, researchers speculate that Piezo2 may also operate through a similar curvature-based gating mechanism. Of course, this also requires further determination of the flat structure of the Piezo2 protein to verify the speculation. The curvature-based gating mechanism of Piezo is shown in Fig. 1 as follows.

#### 2.2.2 Force-from-filament/tether-associated modulation of Piezo gating

In addition to the force-from-lipids mechanism mediated by lipid bilayer tension and membrane curvature, Piezo channels may also be regulated by traction forces related to the cytoskeleton and extracellular matrix in specific cellular environments.^108^^,^^109^ For Piezo1, there is evidence suggesting that it can be directly activated by membrane tension in a cell-free lipid membrane system, indicating that exogenous tethering is not a necessary condition for the activation of Piezo1; however, extracellular matrix proteins can enhance the response of Piezo1 to traction forces, and the E-cadherin-β-catenin-F-actin complex may also connect the Piezo channel to the actin cytoskeleton, thereby regulating the mechanosensitive current of Piezo1.^108^^,^^109^ For Piezo2, its sensitivity to membrane stretching or pressure-clamp stimulation is lower than that of Piezo1, suggesting that Piezo2 may be more dependent on the cytoskeleton, auxiliary connection proteins, or tether-related mechanisms for mechanical gating.^108^^,^^109^ Moreover, the tether structure does not necessarily have to directly pull the channel itself like a “spring”; it may also indirectly affect the opening of the Piezo channel by changing local membrane tension, membrane bending stiffness, or the mechanical state of the cytoskeleton-membrane complex.^108^ Therefore, the force-from-filament/tether-associated mechanism should be regarded as a supplementary regulatory mechanism for Piezo mechanical gating, rather than a completely independent or mutually exclusive process from the force-from-lipids mechanism.^108^^,^^109^

#### 2.2.3 Lever-like mechanotransduction mechanism of Piezo

The force-from-lipids and force-from-filament models describe how external mechanical forces are delivered to Piezo channels, whereas the lever-like mechanism explains how these forces are further transmitted within the Piezo protein structure to open the ion-conducting pore. The transmembrane helical units (THUs) and the central hole serve as the two ends of the lever, while L1342 and L1345 act as the fulcrums as illustrated in Fig. 1A. The fulcrum is positioned close to the end of the central hole. Through this lever-like device, the input force can be amplified, and mechanical force can be transmitted to the center hole, thereby opening the side channel and converting the force into the force used for cation conduction.^15^^,^^111^ The conformational change of the distal transmembrane helical unit (THUs) causes a relatively smaller opening at the center hole end, thereby achieving selective cation conduction as illustrated in Fig. 1B.^115^^,^^116^ The gating mechanisms of many mechanically sensitive ion channels are explained through the lever transduction mechanism, such as TRPA1, TRPV4, K2P, etc..^111^ The Lever-like mechanotransduction mechanism of Piezo is shown in Fig. 1 as follows.

#### 2.2.4 Inactivation of Piezo

The Piezo channel will open the channel when it is stimulated mechanically. After the conduction process is completed, the Piezo will rapidly deactivate and close the channel, which is a significant characteristic of the Piezo channel.^73^ Some researchers have discovered that on the inner helical structure of the Piezo1 and Piezo2, there are two hydrophobic amino acid residues, namely leucine L2475 and valine V2476. This study suggests that the Piezo channel mainly achieves rapid deactivation through the hydrophobic contraction region composed of these two amino acids.^73^ The deactivation kinetics of Piezo1 and Piezo2 are different. In comparison, the deactivation rate of the Piezo1 for the injected current under a given voltage is slower than that of the Piezo2, that is, the deactivation rate of the Piezo1 channel is slower.^66^^,^^74^^,^^75^ Previous studies have found that applying a local magnetic stretching force perpendicular to the cell membrane on the cap structure of the Piezo1 protein can significantly inhibit the deactivation phenomenon of the Piezo1.^74^^,^^117^ It has been reported that Piezo2 can also be activated by membrane stretching, but surprisingly, this study found that the current induced by stretching of Piezo2 has slower deactivation parameters compared to Piezo1, which is contrary to the above-mentioned study results induced by injection.^118^ Therefore, more research in this area is still needed in the future to further explore.

## 3 Functional roles and molecular mechanisms of Piezo-mediated bioelectromagnetic signaling in physiological systems

### 3.1 Conceptual framework of Piezo-mediated bioelectromagnetic activation

Although the term “Piezo-mediated bioelectromagnetic activation mechanism” has not yet been uniformly adopted as a standardized term, it can be used in this review as an integrative conceptual framework. Existing studies support a mechanistic axis in which mechanical or biophysical stimuli activate Piezo channels through lipid-bilayer tension, membrane deformation, or filament/tether-associated force transmission, leading to cation influx, especially Ca^2+^ influx, and electrical or electrochemical signaling.^108^^,^^109^ The subsequent molecular signaling events and tissue-specific physiological responses are further supported by the system-specific studies summarized below^18^^,^^53^^,^^54^^,^^64^^,^^66^ Mechanical or biophysical stimuli, including hydrostatic pressure, shear stress and hemodynamic force, stretch- or pressure-related stimuli, matrix stiffness, interstitial tension, elasticity, and ultrasound stimulation, can activate Piezo1 or Piezo2 in a tissue-specific manner 18, 19, 20, 21, 22, 23^,^^38^, ^39^, ^40^, ^41^, ^42^, ^43^, ^44^, ^45^, ^46^, ^47^, ^48^, ^49^, ^50^, ^51^, ^52^^,^^55^, ^56^, ^57^, ^58^, ^59^, ^60^^,^^119^. Once activated, Piezo channels convert mechanical inputs into electrical, bioelectrochemical, or electrochemical signals mainly through Ca^2+^ influx and related ion-flow events, thereby linking local mechanical environments to downstream biochemical pathways ^21^^,^^35^, ^36^, ^37^, ^38^, ^39^, ^40^, ^41^^,^^54^^,^^55^^,^^57^, ^58^, ^59^, ^60^, ^61^, ^62^, ^63^, ^64^. In the skeletal system, Piezo1-mediated mechanical sensing activates p38 MAPK and ERK1/2 signaling and promotes BMP2, Runx2, Osterix, and ALP expression during osteogenic differentiation 18, 19, 20. In the cardiovascular system, shear-stress-induced Piezo1 activation is associated with nitric oxide synthesis, vasodilation, endothelial homeostasis, and blood pressure regulation 21, 22, 23. In immune cells, Piezo1-dependent Ca^2+^ influx regulates Nox4, HIF1α, NETosis, macrophage phagocytosis, inflammatory responses, and antigen discrimination in B cells ^52^^,^^55^, ^56^, ^57^, ^58^, ^59^, ^60^, ^61^, ^62^. In epithelial organs, Piezo activation further mediates gastrin secretion, mucin2 expression, serotonin release, substance P and peptide YY release, and ATP release, thereby regulating gastric acid secretion, intestinal barrier homeostasis, intestinal motility, urination reflexes, and organ homeostasis 30, 31, 32, 33, 34, 35, 36, 37, 38, 39, 40, 41, 42, 43, 44. Therefore, Piezo-mediated bioelectromagnetic signaling should be understood not only as a channel-opening event, but also as a molecular signaling hub that couples mechanical force to tissue-specific physiological regulation^18^^,^^54^, ^55^, ^56^, ^57^, ^58^, ^59^, ^60^, ^61^, ^62^, ^63^, ^64^

### 3.2 Skeletal system

**Fig. 3:**
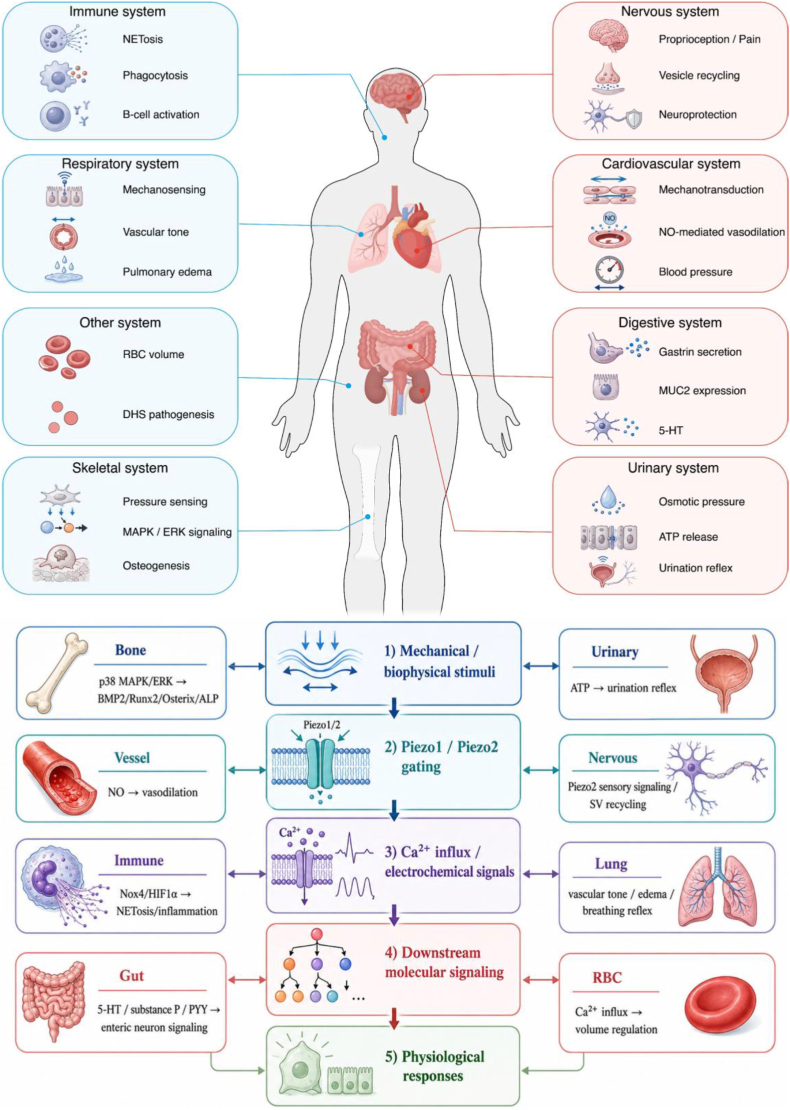
The main role of Piezo in each part and conceptual framework of Piezo-mediated bioelectromagnetic signaling in physiological systems. NETosis: Neutrophil extracellular traposis‌; RBC: Red blood cells; DHS: Dehydrated hereditary stomatocytosis; MUC2: Mucin2 expression (goblet cells); 5-HT: 5-hydroxytryptamine (serotonin); PYY: peptide YY; SV: synaptic vesicles.

The skeleton, as an actively metabolizing organ in vertebrates, continuously undergoes repair, regeneration and remodeling throughout its lifetime.^16^ J.L. Wolff proposed that the skeleton can respond to mechanical and biophysical stimuli, and daily activities such as movement and mechanical operations can impose mechanical loads on the skeleton.^17^ Moreover, mechanical loads can regulate the biological functions of bone tissue through mechanical sensors within bone cells, thereby promoting fracture healing and bone regeneration.^17^ Piezo1 plays the role of a hydrostatic pressure sensor in bone marrow mesenchymal stem cells (BMSCs), and a hydrostatic pressure of 0.01 MPa can activate the p38 mitogen-activated protein kinase (MAPK) and extracellular signal-regulated kinase 1/2 (ERK1/2) signaling pathways through the Piezo1, thereby inducing the expression of the growth factor BMP2 that promotes the differentiation of BMSCs into osteoblasts. At the same time, this will also promote the generation of Runx2 and Osterix, initiating the expression of osteogenic-related genes such as ALP, thereby promoting the differentiation of BMSCs into osteoblasts and inhibiting their differentiation into adipocytes as illustrated in Fig. 3 18, 19, 20. Mechanistically, Piezo1 links hydrostatic pressure and mechanical loading to osteogenic molecular signaling in BMSCs. This process can be summarized as hydrostatic pressure–Piezo1 activation–p38 MAPK/ERK1/2 signaling–BMP2 expression–Runx2/Osterix activation–ALP-related osteogenic gene expression, ultimately promoting osteoblast differentiation and bone regeneration 18, 19, 20. Therefore, in the skeletal system, Piezo1 may act as a mechanosensitive converter that connects mechanical loading with osteogenic biochemical signaling.

### 3.3 Cardiovascular system

The Piezo1 ion channel is abundantly present in the capillary endothelial cells of the brain and may have the function of regulating cerebral blood flow. The realization of this function may be achieved through the mechanical deformation of capillary endothelial cells and the shear stress of blood flow when red blood cells are transported within capillaries, thereby opening the Piezo1 channel, converting mechanical signals into electrical signals and regulating lower tract metabolic activities as illustrated in Fig. 3.^21^ Generally, the integrity of vascular endothelial cells is mainly maintained by the dynamic balance between hemodynamic effects and biological responses. Blood flow disturbances caused by hypertension can lead to vascular dysfunction.^22^ Piezo1 can sense and convert hemodynamic forces, thereby maintaining vascular function and the homeostasis of vascular endothelial cells.^22^ Systemic hypertension is regulated by many mechanical signals, including vasodilation caused by nitric oxide.^23^ Piezo can induce nitric oxide synthesis and vasodilation induced by shear stress, and it can also regulate the reflex activities of the aorta and carotid artery. Therefore, Piezo plays a crucial role in blood pressure control.^23^ In the cardiovascular system, Piezo1-mediated signaling mainly reflects the conversion of hemodynamic force into endothelial electrical and biochemical responses. Blood-flow-induced shear stress can activate Piezo1 in endothelial cells, thereby linking vascular mechanical stimulation to nitric oxide synthesis, vasodilation, endothelial homeostasis, and blood pressure regulation 21, 22, 23. These findings suggest that Piezo1 serves as an important mechanosensitive interface between vascular mechanical force and bioelectrical regulation.

### 3.4 Nervous system

Proprioception refers to the ability to sense the position of our limbs in space, which enables us to perform a series of movements mainly through the sensory feedback generated by the musculoskeletal system.^24^ Studies have shown that Piezo2 is the main mechanotransduction ion channel for human proprioception. When Piezo2 undergoes mutations, patients may experience movement disorders and sensory ataxia due to their inability to perceive the position of their limbs.^25^ Piezo2 signaling is associated with functional defects and pain behaviors in osteoarthritis, musculoskeletal pain, distal arthrogryposis, Angelman syndrome, and arterial stiffness as illustrated in Fig. 3 26, 27, 28, 29. Researchers conducted next-generation sequencing analysis on four generations of family members with distal arthrogryposis, and found that affected newborns and their mothers had pathogenic heterozygous mutations in Piezo2.^26^ Studies have shown that inducing a mutation in the proprioceptive neurons that mainly innervate the muscles and tendons of mice, which enhances the expression of Piezo2, leads to distal joint contractures in mice, indicating that excessive mechanical sensing within neurons can disrupt the normal development of the musculoskeletal system.^27^ In Angelman syndrome patients, the absence of UBE3A expression on the maternal chromosome is the main cause of this neurogenetic disorder, as the loss of UBE3A reduces the expression and function of Piezo2.^28^ During inflammation, sensory neurons that originally prevent tissue damage, namely nociceptors, become hypersensitive and respond to harmless stimuli. Research indicates that mechanical pain in osteoarthritis is related to Piezo2, and the sensitization of joint nociceptors mediated by neurotrophic factors is associated with Piezo2.^29^

The accumulation of α-synuclein (α-syn) in the nervous system leads to the disruption of the synaptic vesicle (SV) cycle, thereby exacerbating the difficulty of normal dopamine release and the degeneration of dopaminergic neurons in the substantia nigra pars compacta (SNc), which are the main causes of the occurrence of neurodegenerative diseases such as Parkinson's disease.^120^ Excessive Fe^2+^ not only promotes the accumulation of α-synuclein (α-syn) by binding with it to form insoluble substances, but also can oxidize dopamine into neurotoxins. Therefore, ferroptosis is considered a new target for the treatment of Parkinson's disease.^121^^,^^122^ Studies have shown that optimized ultrasound stimulation can promote the recycling of synaptic vesicles (SV) by activating the endocytosis mediated by the Piezo1, thereby reducing the accumulation of α-synuclein (α-syn) and inhibiting the exacerbation of ferroptosis, thus protecting the nerves and treating Parkinson's disease. This provides a new idea for biological electromagnetic intervention therapy.^119^ In the nervous system, Piezo2-mediated mechanotransduction converts mechanical stimuli into sensory signals required for proprioception and mechanically evoked pain responses 24, 25, 26, 27, 28, 29. In addition, ultrasound-induced Piezo1 activation has been reported to promote synaptic vesicle recycling, reduce α-synuclein accumulation, and inhibit ferroptosis-related neurodegeneration, suggesting a potential Piezo-related mechanism for bioelectromagnetic intervention in neuronal protection 119, 120, 121, 122. However, this intervention-related mechanism should be distinguished from the classical Piezo2-mediated sensory mechanotransduction pathway.

### 3.5 Immune system

Piezo1 regulates downstream signaling pathways by controlling the intracellular and extracellular Ca^2+^ concentrations. It is present in various immune cells and has a significant impact on immune function as illustrated in Fig. 3.^55^ Neutrophils are the main immune cells present in the blood. During their journey to the infection site upon receiving signals, they are subjected to mechanical pressure from the blood vessel walls, thereby activating the Piezo1, resulting in Ca^2+^ influx, enhanced expression of NADPH oxidase 4 (Nox4) and stable hypoxia inducible factor 1α (HIF1α), and expression of neutrophil elastase, forming neutrophil extracellular traps (NETosis), ultimately enhancing their ability to engulf bacteria.^55^ The mononuclear phagocytic complex is a composite structure derived from monocytes and their precursor cells in the bone marrow. During its journey to the infection site in the lungs to carry out the immune response, it will be subjected to the static water pressure and shear stress of the blood, thereby activating the Piezo channels and CD11b and generating endothelin-1, stable hypoxia inducible factor 1α (HIF1α), and pro-inflammatory factors, enhancing the adhesion of endothelial cells to monocytes, the phagocytic activity of macrophages derived from monocytes, and the uptake of oxidized low-density lipoprotein 52,56. Macrophages are present in various tissues and organs, and their Piezo1 can be activated by external mechanical forces such as matrix hardness, interstitial tension, and elasticity, causing intracellular Ca^2+^ influx. At the same time, under the action of chemical stimuli, it responds differently depending on the immune state 57, 58, 59. Studies have shown that by knocking out the Piezo1 gene in mice, the phagocytic ability, bacterial clearance ability, and the content of reactive oxygen species in mitochondria and phagosomes of mouse macrophages were detected, indicating that Piezo1 is a key regulatory factor for macrophages to exert the functions of clearing bacteria and phagocytosis.^59^ Some researchers have found that macrophages can differentiate into two phenotypes, pro-inflammatory M1 and anti-inflammatory M2, depending on the hardness of the extracellular matrix. In softer matrices, Piezo1 causes macrophages to differentiate into a round M1 phenotype with lower adhesion and migration abilities, which increases the inflammatory response to resist pathogenic bacteria.^60^ B cells and T cells are both immune cells derived from bone marrow hematopoietic stem cells. After being stimulated by antigens, they can release immunoglobulins to participate in humoral immunity. B cells distinguish soluble antigens from membrane-presenting antigens by the influx of Ca^2+^ through the Piezo1, and then produce a more rapid and efficient response.^61^^,^^62^ In addition, Dendritic cells, Neuroglial cells, and Natural Killer cells also require the regulation and participation of Piezo1 during immune responses. Mechanistically, Piezo1-mediated immune regulation is closely associated with Ca^2+^-dependent inflammatory signaling. Mechanical pressure, matrix stiffness, interstitial tension, or elasticity can activate Piezo1 in neutrophils and macrophages, inducing Ca^2+^ influx and promoting Nox4, HIF1α, NETosis, macrophage phagocytosis, bacterial clearance, and inflammatory responses ^52^^,^^55^, ^56^, ^57^, ^58^, ^59^, ^60^. In B cells, Piezo1-mediated Ca^2+^ influx further helps distinguish soluble antigens from membrane-presenting antigens, thereby improving immune recognition efficiency.^61^^,^^62^ These findings indicate that Piezo1 functions as a mechanical-to-calcium signaling interface in immune-cell activation.

### 3.6 Digestive system

The gastrointestinal tract needs to sense and adapt to various mechanical stimuli in order to complete the functions of digesting and absorbing food and resisting foreign pathogens and toxins.^30^ Studies have found that Piezo1 is involved in the regulation of gastrointestinal functions and mechanical sensation. The Piezo1 is precisely located in the basal lateral part of the G cells in the gastric antrum that release gastrin (a hormone that controls gastric activity), so the Piezo1 may be affected by changes in food pressure on the gastric wall, secrete gastrin, and thereby increase gastric acid secretion.^31^^,^^32^ Research has reported that the Piezo1 protein in goblet cells is stimulated by mechanical stimuli, which promotes the expression of mucin2 (mucin 2) in goblet cells, and mice with Piezo1 defects have intestinal flora imbalance, changes in mucin2, and mild inflammatory states. These findings indicate that the Piezo1 in goblet cells plays a crucial role in balancing intestinal flora and homeostasis, and it also responds to mechanical stimuli to maintain intestinal barrier function.^33^^,^^34^ The Piezo2 plays a key role in the mechanical sensitivity of intestinal epithelial cells and the release of serotonin. Activation of the Piezo2 in intestinal epithelial cells causes Ca^2+^ influx, resulting in an increase in intracellular Ca^2+^ concentration, which leads to mechanosensitive serotonin release. Additionally, knocking out the Piezo2 or using drugs to inhibit its expression will reduce the amount of serotonin release and the physiological effects of mechanosensitive currents and downstream reactions.^35^^,^^36^ Studies have found that Piezo2 in intestinal epithelial cells can sense the amount of substances in the intestinal lumen. After the Piezo2 is activated, it not only releases serotonin, substance P, and peptide YY, but also transmits bioelectrochemical signals to the intestinal neurons responsible for peristalsis and secretion, thereby regulating the peristalsis of the entire intestine as illustrated in Fig. 3.^36^^,^^37^ In the digestive system, Piezo-mediated signaling is mainly reflected in mechanically induced hormone and neurotransmitter release. The Piezo1 may sense gastric wall pressure in G cells and regulate gastrin secretion, while the Piezo1 in goblet cells contributes to mucin2 expression and intestinal barrier homeostasis 31, 32, 33, 34. The Piezo2 activation in intestinal epithelial cells induces Ca^2+^ influx and promotes serotonin release, substance P and peptide YY secretion, and bioelectrochemical communication with enteric neurons, thereby regulating intestinal peristalsis and secretion 35, 36, 37. Thus, Piezo channels may couple gastrointestinal mechanical stimulation to secretory, neural, and barrier-regulatory responses.

### 3.7 Urinary system

Studies have reported that when the Piezo1 gene in the renal epithelial cells of mice is knocked out, the mice will experience dehydration or a decrease in urea concentration after fasting, as well as impaired urine dilution ability. This indicates that Piezo1 exists in the kidneys and regulates urine osmotic pressure as illustrated in Fig. 3.^38^ The Piezo1 in renal tubular epithelial cells can interact with polycystin-2 and its mutant PC2-740X, inhibiting the mechanical sensitivity of Piezo1, thereby regulating the internal pressure in the kidneys.^39^ Piezo1 and Piezo2 are crucial for urination. In urinary epithelial cells, Piezo1 undergoes Ca^2+^ influx and releases ATP after being stimulated by stretching. Piezo2 makes the lower urinary tract sensitive to stretching stimuli, thereby regulating the urination reflex.^40^^,^^41^ In mice with Piezo1 gene knockout but still able to urinate normally, the amount of ATP released by urinary epithelial cells did not significantly decrease, possibly due to the existence of potential compensatory mechanisms to overcome the absence of Piezo1.^42^ The ATP release mediated by Piezo1 and Piezo2 in urinary epithelial cells is very important for the urination process. The Piezo1 and TRPV4 mechanosensitive ion channels in urinary epithelial cells are time-dependent, so urinary epithelial cells can sense the diurnal pattern of bladder filling. The expression of mechanosensitive ion channels increases during the day and decreases during the sleep period at night. Moreover, experiments have found that in mice with clock gene mutations, the expression of Piezo1 in the bladder is constant.^43^^,^^44^ In the urinary system, Piezo-mediated mechanotransduction mainly couples stretch stimulation to Ca^2+^ influx and ATP release. Stretch-induced Piezo1 activation in urinary epithelial cells promotes Ca^2+^ influx and ATP release, while Piezo2 contributes to lower urinary tract stretch sensitivity and urination reflex regulation.^40^^,^^41^ The time-dependent expression of Piezo1 and TRPV4 further suggests that mechanosensitive ion channels may integrate bladder filling with circadian regulation of urinary function.^43^^,^^44^

### 3.8 Respiratory system

The Piezo1 is present in pulmonary endothelial cells and pulmonary epithelial cells. It can regulate cell arrangement and division activities in pulmonary epithelial cells, and it can inhibit pulmonary vascular contraction in pulmonary vessels.^45^^,^^66^ Studies have found that Piezo1 does not affect blood pressure when not in motion, but it causes blood pressure to rise during activity as illustrated in Fig. 3. This may be because the increase in blood flow during exercise activates the Piezo1 in vascular smooth muscle cells, thereby causing vascular contraction and affecting blood pressure.^46^ Additionally, compared to normal individuals, the expression of Piezo1 in pulmonary endothelial cells of patients with pulmonary hypertension is significantly increased.^47^ Pulmonary endothelial cells with the Piezo1 are stimulated by increased pressure in pulmonary microvessels and can trigger pulmonary edema.^53^ By knocking out the Piezo1 in mouse pulmonary endothelial cells and increasing pulmonary microvascular pressure, it was found that mice did not show symptoms of pulmonary edema. Thus, the Piezo1 is a key factor in the pathogenesis of pulmonary edema.^53^ The Piezo1 is also closely related to non-small cell lung cancer. Studies have reported that the mRNA expression of Piezo1 in lung tissue of non-small cell lung cancer patients is significantly lower than that in normal tissue, which may be attributed to the fact that the Piezo can regulate cell migration and tumor growth.^48^ Some researchers have found that knocking out Piezo1 in mouse myeloid cells can alleviate the inflammatory symptoms of mice after bacterial infection, and mice without Piezo1 have significantly increased susceptibility to *Pseudomonas aeruginosa*. Moreover, monocytes and macrophages respond to mechanical stimulation of the Piezo1 in vitro and cause inflammatory reactions.^52^ Defects in the Piezo2 production can affect the respiratory function of newborns and they always have shallow and rapid breathing throughout their lives. Additionally, studies have found that mice lacking the Piezo2 in cervical nerve-olfactory nerve ganglia, trigeminal nerve, and spinal nerve ganglia will die within 24 h after birth due to respiratory failure.^49^^,^^50^ Knocking out the Piezo2 in adult mouse sensory neurons, as well as its abnormal activation, leads to impairment of the Hering-Breuer reflex and respiratory system problems.^50^^,^^51^ In the respiratory system, Piezo-mediated signaling links pulmonary mechanical stress to vascular, epithelial, and reflexive responses. The Piezo1 in pulmonary endothelial and epithelial cells participates in cell arrangement, pulmonary vascular tone, pulmonary hypertension, and pressure-induced pulmonary edema 45, 46, 47, 48^,^^53^^,^^66^. The Piezo2 is also required for normal respiratory mechanoreflexes, as its defects impair neonatal breathing and the Hering–Breuer reflex 49, 50, 51. These findings suggest that the Piezo1/2 contribute to respiratory mechanoregulation through both cellular and neural mechanisms.

### 3.9 Other systems

The Piezo1 contributes to the stability of red blood cells and is a major determinant of red blood cell volume regulation as illustrated in Fig. 3. Studies have shown that if the Piezo1 gene in red blood cells is knocked out, resulting in the inability of Piezo1 to be expressed in red blood cells, then the red blood cells will absorb excessive water and be prone to bursting.^63^ Dehydrated hereditary oral erythrocytosis (DHS) is a human congenital hemolytic disease. Patients with this disease will experience dehydration symptoms in their red blood cells. The reason for this is the overexpression mutation of the Piezo1. By comparing it with the wild-type channel, it was found that these mutated Piezo1 would have an intensified influx of Ca^2+^ and enhanced electrochemical signals under mechanical stimulation.^54^^,^^64^ The distribution diagram of the biological signals mediated by the Piezo in our human body is shown as Fig. 3 below. In red blood cells, Piezo1-mediated signaling is closely related to volume regulation and electrochemical homeostasis. Loss of Piezo1 expression impairs red blood cell stability, whereas gain-of-function Piezo1 mutations enhance Ca^2+^ influx and electrochemical signals under mechanical stimulation, leading to abnormal red-blood-cell dehydration and volume regulation.^54^^,^^63^^,^^64^ This provides another example of how Piezo-mediated ion flux can convert mechanical deformation into physiologically relevant electrochemical responses.

### 3.10 Perspectives on Piezo-mediated bioelectromagnetic modulation

Overall, the studies summarized above suggest that Piezo-mediated bioelectromagnetic modulation is not a single uniform pathway, but a context-dependent mechanotransduction framework. In different physiological environments, the Piezo1 and Piezo2 respond to distinct mechanical or biophysical cues, including hydrostatic pressure, shear stress, stretch, matrix stiffness, tissue deformation, and ultrasound-related force. These stimuli are converted into ion flux, especially Ca^2+^ influx, electrochemical signals, and downstream molecular responses.

However, the strength of evidence varies among physiological systems. Relatively clear downstream mechanisms have been reported in skeletal, cardiovascular, immune, gastrointestinal, urinary, respiratory, and red blood cell systems, whereas the direct causal relationship connecting external physical-field interventions, Piezo activation, and downstream tissue-level outcomes warrants further investigation. Future studies should combine electrophysiology, Ca^2+^ imaging, Piezo-specific inhibitors, genetic knockout models, and well-defined physical stimulation parameters to clarify the causal mechanisms of Piezo-mediated bioelectromagnetic modulation.

## 4 Piezo's association with diseases and physical therapy intervention strategies

### 4.1 The association between Piezo and diseases

Mutations in the Piezo gene can lead to the occurrence of various human genetic diseases. For instance, activation mutations and inactivation mutations in Piezo1 can respectively cause hemolytic anemia, hereditary oral cell hyperplasia, and hereditary lymphatic malformation; while activation mutations and inactivation mutations in Piezo2 can respectively result in hereditary distal joint contracture syndrome and type 1 muscular atrophy with perinatal respiratory distress, joint contracture, and scoliosis.^123^ Abnormal expression of the Piezo protein is closely related to hypertrophic obstructive cardiomyopathy and osteoporosis. The expression level of Piezo1 in cardiomyocytes of patients with cardiomyopathy is significantly increased, while the expression level of Piezo1 in patients with osteoporosis is significantly decreased. Moreover, the expression level of the *Piezo* gene in human bone marrow mesenchymal stem cells decreases with age 124, 125, 126.

The Piezo is also closely related to various types of cancers. The expression of Piezo1 is significantly upregulated in the tissues of colon cancer patients, and the Piezo1 can promote the migration of colon cancer cells in vitro experiments.^127^ Additionally, the expression of Piezo2 is significantly decreased in the tissues of breast cancer patients, and the expression of Piezo1 is also significantly downregulated in the tissues of non-small cell lung cancer patients.^48^^,^^128^ Numerous studies have shown that the Piezo1 is a potential new target for cancer immunotherapy.^129^^,^^130^ The lack of Piezo1 will impair the differentiation of TH9 cells, thereby reducing the cytotoxicity of immune cells T cells and enhancing the activity of regulatory T cells Tregs. The Piezo1 promotes immunity in tumor cells by regulating processes such as cancer-related fibroblasts and extracellular matrix remodeling, and promotes the proliferation and metastasis of cancer cells by activating oncogenic signals.^129^ Studies have found that after the Piezo1 is stimulated by mechanical forces, it inhibits the expression of the retinoblastoma gene Rb1 to promote the expansion of myeloid suppressor cells. When the Piezo1 is inhibited, it can effectively prevent cancer and also reduce myeloid suppressor cells, and knocking out the Piezo1 in myeloid cells can also prevent cancer.^130^ Another study shows that the Piezo1 and integrin β1 (ITGB1) accelerate the development of bladder cancer by promoting cell proliferation and inhibiting cell apoptosis.^131^ The Piezo1 and ITGB1 can sense the hardness of the extracellular matrix, and the hardness of the extracellular matrix activates the synergistic effect of Piezo1/ITGB1 to generate Ca^2+^ influx, thereby promoting the nuclear translocation of YAP, upregulating the targets CTGF, α-SMA and COL1A1 of the downstream signaling pathway, thereby strengthening collagen deposition and extracellular matrix hardening, and accelerating the progression of bladder cancer.^131^

The cardiac functions related to the Piezo channels include the conversion of hemodynamic forces in endothelial cells and vascular cells, erythrocyte homeostasis, platelet aggregation, and arterial blood pressure regulation, etc..^63^ The Piezo1 is present in the cell membranes of endothelial cells and the intercellular junction complexes, and it can sense mechanical stimuli brought by blood flow and convert mechanical forces into biochemical signals to regulate the homeostasis and vascular function of endothelial cells.^132^ In endothelial cells, the Piezo1 also regulates the formation and shaping process of cell adhesion connections, the process of white blood cell extravasation from blood vessels, and the opening of the TRPV4 channel.^133^^,^^134^ The Piezo channels are also associated with pathological conditions, including myocardial hypertrophy and pulmonary arterial hypertension, etc..^63^ In mice, the Piezo1 is expressed in endothelial cells and endocardium from day 9 to 10.5 of mouse embryonic development, but if the Piezo1 remains activated continuously, it will cause a large amount of pericardial effusion, undeveloped main blood vessels, and disordered vascular structure in mice, ultimately leading to the death of mice during pregnancy. Moreover, if the Piezo1 in mouse endothelial cells is specifically inhibited or inactivated, it will result in impaired lymphatic valve formation or maldevelopment of the aortic valve in mice.^132^^,^^135^^,^^136^ The Piezo1 in endothelial cells are activated when the pressure in pulmonary microvessels increases, which leads to the destruction of the calcium protease on vascular endothelial-cadherin adhesion, thereby causing the disruption of the endothelial barrier and triggering pulmonary edema.^53^ As the Piezo1 acts as an initiator of many mechanosensitive signaling pathways, a growing number of studies have demonstrated its crucial importance in cardiovascular development.

Osteoarthritis is a disease that causes the degradation of cartilage, inflammation, and stiffness and pain in patients, and it can also lead to lifelong disability.^137^ The research has found that in the joint cartilage cells of patients and mice with osteoarthritis, abnormal mechanical load or inflammatory cytokines can significantly increase the expression level of Piezo1, leading to an increase in the senescence and apoptosis of cartilage cells, thereby accelerating the erosion of cartilage. When the Piezo1 gene in the joint cartilage cells of mice is knocked out, it significantly alleviates the symptoms of osteoarthritis in mice.^137^^,^^138^ Moreover, the study also indicates that injecting Yoda1 into the mice's joints further activates the Piezo1, thereby exacerbating the symptoms of osteoarthritis in mice, while silencing the Piezo1 gene using siRNA technology reduces the symptoms of osteoarthritis in mice.^138^ Some researchers have discovered that the Piezo1 coordinates the interaction between immune Th17 cells and mesenchymal stem cells by receiving mechanical load stimulation, thereby regulating the physiological process of bone remodeling in osteoarthritis.^139^ Additionally, the experimental results of this study also support the hypothesis that bone-forming cells need the Piezo1 to sense mechanical load and regulate bone metabolic activities.^139^ Moreover, the Piezo1 can promote the expression of enzymes that break down the components of the cartilage matrix, such as matrix metalloproteinases (MMPs) and aggrecanases, thereby exacerbating the destruction of the cartilage structure.^137^ Thus, the Piezo1 plays a crucial regulatory role in the progression of osteoarthritis and demonstrates its potential as a therapeutic target in the future. The association between the Piezo and diseases is shown in Table 1, Table 2.

**Table 1:** The Piezo1 and human diseases.

System Name	Disease/Symptom	Mechanics	Reference
Skeletal system	Bone trauma	In bone marrow mesenchymal stem cells, Piezo1, when stimulated by hydrostatic pressure, activates the p38 mitogen-activated protein kinase (MAPK) and extracellular signal-regulated kinase 1/2 (ERK1/2) signaling pathways, promoting the production of growth factors BMP2, Runx2 and Osterix, and initiating the expression of the osteogenic gene ALP.	18, 19, 20
Cardiovascular system	Generalized hypertension	The Piezo1 protein in vascular endothelial cells is unable to sense mechanical deformation and shear stress, and thus cannot regulate downstream metabolic activities.	21, 22, 23
Nervous system	Parkinson's disease	Enhanced expression of Piezo1 promotes synaptic vesicle (SV) recycling, reduces the accumulation of α-synuclein (α-syn), and decreases ferroptosis in neurons.	119
Immune system	Weakened immunity	The Piezo1 protein of neutrophils is subjected to pressure from the vessel wall during transportation, thereby regulating the expression of NADPH oxidase 4 (Nox4) and stable hypoxia inducible factor 1α (HIF1α), Neutrophil (NEUT) elastase and thereby affecting the phagocytic ability of neutrophils.	55
		The Piezo and CD11b proteins of the mononuclear phagocytic complex are activated during transportation by hydrostatic pressure and shear stress, resulting in the production of endothelin-1, stable hypoxia-inducible factor 1α (HIF1α), and pro-inflammatory factors.	52,56
		The Piezo1 protein in macrophages can differentiate into two different phenotypes depending on the type of extracellular matrix. In soft matrix conditions, macrophages will differentiate into the pro-inflammatory M1 type, increasing the inflammatory response to combat pathogenic bacteria.	60
		B cells, through the Piezo1, allow Ca^2+^ to flow in, which distinguishes between soluble antigens and membrane-presenting antigens, thereby triggering a more rapid and efficient immune response.	61,62
Digestive system	Poor digestion and absorption	In the G cells of the gastric antrum, Piezo1 will be activated upon stimulation by the pressure of food on the gastric wall. This leads to the secretion of gastrin, which promotes the secretion of gastric acid that can digest food.	31,32
	Imbalance of intestinal flora	The Piezo1 in goblet cells is activated by mechanical stimulation and promotes the expression of mucin 2.	33,34
Urinary system	Disruption of urine osmotic pressure	In renal tubular epithelial cells, Piezo1 can interact with polycystin-2 and its mutant PC2-740X, inhibiting the mechanical sensitivity of Piezo1 and regulating the osmotic pressure of urine.	39
	Inability to urinate	The Piezo1 is present in urothelial cells, and when it is stretched, it causes Ca^2+^ influx and ATP release, thereby regulating the urination reflex.	40,41
	Irregular urination	The Piezo1 in urothelial cells is time-dependent; its expression increases during the day and decreases at night.	43,44
Respiration system	Pulmonary arterial hypertension	In the pulmonary endothelial cells of patients with pulmonary hypertension, the expression of Piezo1 is significantly increased; during exercise, the Piezo1 in vascular smooth muscle cells are activated due to the increase in blood flow, causing vascular contraction.	46,47
	Abnormal inflammatory response	The absence of Piezo1 in mouse myeloid cells causes an increase in susceptibility to *Pseudomonas aeruginosa* and alleviates the symptoms of mice infected by bacteria. The mechanical stimulation of monocytes and macrophages on Piezo1 causes inflammatory responses in vitro.	52
	Pulmonary edema	The Piezo1 is a key factor in the pathogenesis of pulmonary edema. The Piezo1 in urothelial cells of the lungs is stimulated by the increase in pulmonary microvascular pressure and triggers pulmonary edema.	53
Other system	Red blood cells bursting/Hereditary oral erythrocytosis due to dehydration	The absence of Piezo1 in red blood cells leads to abnormal volume regulation, causing them to absorb excessive water and burst; overexpression and mutation of Piezo1 can result in increased Ca^2+^ influx and enhanced electrochemical signals in response to certain mechanical stimuli.	54,63,64

**Table 2:** The Piezo2 and human diseases.

System Name	Disease/Symptom	Mechanics	Reference
Nervous system	Ataxia and sensory ataxia	A mutation in the Piezo2 prevents it from sensing the position of its own limb.	25
	Distal joint contracture syndrome	Enhanced expression of the Piezo2 leads to excessive mechanical sensation by neurons, resulting in abnormal bone development.	26
	Angelman syndrome	The absence of UBE3A expression leads to a decrease in the Piezo2 expression.	28
	Osteoarthritis	The Piezo2 causes sensitization of joint pain receptors.	29
Digestive system	Intestinal constipation	After activation of the Piezo2 in intestinal epithelial cells, it promotes Ca^2+^ influx, increasing intracellular Ca^2+^ concentration and facilitating serotonin release.	35,36
		When the Piezo2 in intestinal epithelial cells is stimulated by the intestinal lumen, it not only releases serotonin, substance P, and peptide YY, but also transmits biochemical signals to regulate intestinal neurons responsible for peristalsis and secretion, promoting intestinal peristalsis.	36,37
Urinary system	Inability to urinate	The Piezo2 expression in urothelial cells in the lower urinary tract transmits biochemical signals to regulate the urination reflex after being stimulated by stretching.	40,41
Respiration system	Abnormal respiratory function	Abnormal expression of the Piezo2 can cause newborns to have a lifelong phenomenon of shallow and rapid breathing, and can also lead to impaired Hering-Breuer reflex and respiratory system problems in adult mice. Mice lacking the Piezo2 will die within 24 h of birth.	49, 50, 51

### 4.2 Physical-field intervention strategies targeting Piezo-mediated bioelectromagnetic modulation

#### 4.2.1 Ultrasound-based strategies

Ultrasound is a widely used non-invasive physical stimulus with high spatial and temporal controllability and has been explored for Piezo-related modulation due to its ability to generate mechanical forces in biological tissues.^140^ Ultrasound-mediated drug delivery mainly relies on mechanical and thermal effects, including cavitation-induced carrier disruption and temperature-dependent release mechanisms 141, 142, 143. However, these effects are not necessarily mediated by Piezo channels. Currently, nanosystems for drug release include liposomes, microbubbles, and silicon-based nanoparticles. Importantly, ultrasound stimulation can be coupled with piezoelectric biomaterials to generate local bioelectrical signals and directly regulate mechanosensitive ion channels. For example, whitlockite (WH) nanomaterials stimulated by low-intensity pulsed ultrasound generate bioelectric signals that activate Piezo1 and TRPV4 channels, induce Ca^2+^ influx, and promote osteoblast proliferation and differentiation.^144^

In addition to the Piezo activation, ultrasound-responsive piezoelectric semiconductors have also been explored for sonodynamic and catalytic therapies through ROS generation and tumor inhibition 145, 146, 147, 148, 149. However, these therapeutic effects are mainly attributed to material-mediated catalytic reactions, and their direct dependence on Piezo-channel activation remains unclear.

Current evidence suggests that ultrasound-based Piezo modulation mainly acts through mechanical stimulation or piezoelectric-material-generated electrical cues. Among these strategies, the strongest mechanistic evidence is provided by studies demonstrating ultrasound-induced Piezo activation, Ca^2+^ influx, and downstream biological responses.^144^ In contrast, ultrasound-mediated drug delivery and sonodynamic therapy represent broader physical-field applications, and their relationship with Piezo channels requires further clarification.

#### 4.2.2 Magnetic stimulation-based strategies

Magneto-genetics, which includes magneto-mechanical and magneto-thermal modalities, derives its key advantages from the ability of magnetic fields to penetrate biological tissues noninvasively, thereby enabling wireless activation of deep internal targets. Magneto-mechanical-genetics utilizes the magnetic field to generate mechanical force. Because it can achieve rapid and reversible ion channel gating without raising the temperature of the surrounding tissues, it has significant advantages over magneto-thermal-genetics when conducting large-scale in vivo research.^150^ Additionally, this research successfully expressed the Piezo1 mechanosensitive ion channel in specific cell types using the Cre-LoxP recombination system‌, and then used magnetic stimulation to regulate specific cell populations of neurons, thereby developing a cell type-specific magnetic stimulation strategy applicable to the deep circuits of the brain in live animals, and this technology can provide long-distance and large-scale neural stimulation.^150^ Magnetic stimulation provides a non-invasive approach for remote mechanical modulation because magnetic forces can be converted into mechanical stimuli capable of regulating mechanosensitive ion channels. A magnetic toolkit containing a circular magnetic array and a nanoscale magnetic torque driver was developed by a research team. It can provide a force of piconewtons within a range of 70 cm, and it can work for several tens of centimeters over a long distance to stimulate the Piezo channels in targeted neuronal cells, thereby achieving non-contact neural regulation.^151^ Hydroxyapatite whisker (HAw) is a synthetic whisker-shaped nano-hydroxyapatite, which is widely used for repairing bone tissues and improving the performance of bioceramics due to its similarity to the components of natural bone tissue. However, it has the drawback of poor bone induction ability 152, 153, 154. Studies have found that a magnetic framework formed by combining iron oxide nanoparticles, superparamagnetism and controllable alignment along the magnetic field direction, with Hydroxyapatite whisker (HAw) can activate the Piezo1-mediated MAPK pathway, thereby significantly accelerating the mineralization of bone matrix.^155^ Research has developed a type of PiezoMagnetic nanoparticles, which is made of piezoelectric barium titanate and superparamagnetic iron oxide nanoparticles. These nanoparticles can respond to magnetic fields, ultrasound waves and light.^156^ They possess excellent electrical conductivity and ferroelectric properties. Under non-invasive low-intensity pulsed ultrasound stimulation, they can generate biological signals resembling those under natural conditions. Therefore, PiezoMagnetic nanoparticles are applied in the treatment of multimodal cancer therapy and non-invasive cell stimulation, such as being able to eliminate osteosarcoma cells. It can also be used in photothermal therapy, magneto-thermal therapy and photodynamic therapy.^156^ Furthermore, by combining PiezoMagnetic nanoparticles with a new generation of bisphosphonates that have the function of inhibiting osteoclasts' ability to absorb bone, when subjected to low-intensity pulsed ultrasound, they can release the bisphosphonates, promoting bone remodeling and regeneration, and thus being used for the repair and treatment of bone damage after osteosarcoma surgery.^156^

Compared with conventional magnetic-field therapy, magneto-mechanical strategies are more directly associated with Piezo-mediated bioelectromagnetic modulation because they convert magnetic inputs into mechanical forces that can regulate Piezo channels. Current studies provide evidence that magnetic stimulation can remotely regulate Piezo1-expressing neurons and activate Piezo1-related signaling in bone regeneration. However, the quantitative relationship between magnetic parameters, mechanical force generation, Piezo gating, and downstream physiological outcomes remains to be established.

#### 4.2.3 Piezoelectric material-based strategies

**Fig. 4:**
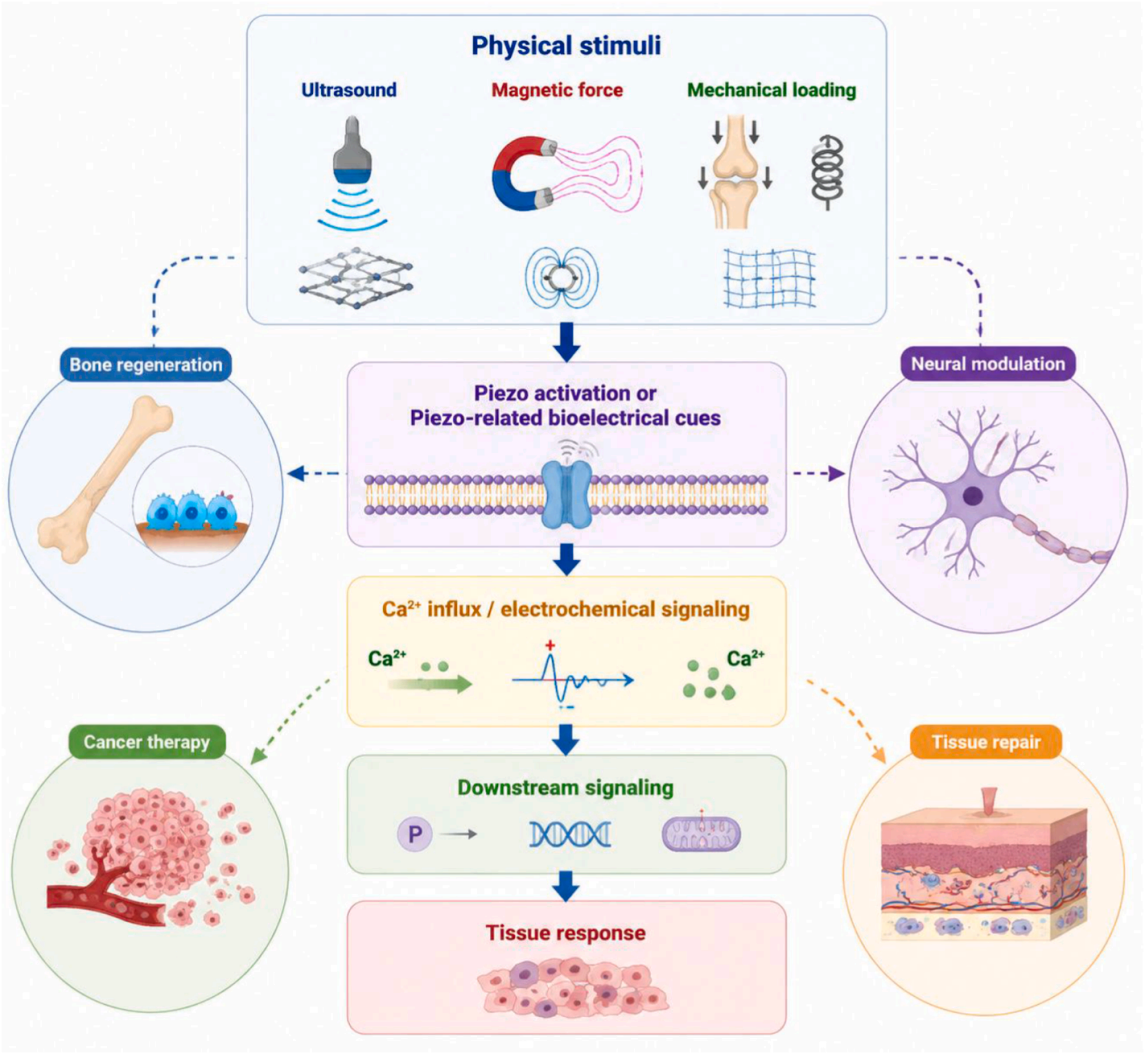
Overview of physical-field strategies for Piezo-mediated bioelectromagnetic modulation and their physiological applications.

Piezoelectric biomaterials provide a unique strategy for bioelectromagnetic intervention because they can convert mechanical deformation into local electrical stimulation without external power sources.^147^^,^^157^ Importantly, their therapeutic effects may involve both direct electrical stimulation and regulation of mechanosensitive ion channels, including Piezo-related signaling pathways. Researchers have developed a biodegradable piezoelectric polylactic acid nanofiber scaffold that does not require a battery. Compared to non-piezoelectric scaffolds, this scaffold enables the complete healing of rabbit cartilage within 2-3 months after surgery. This is because when the scaffold is subjected to mechanical pressure or joint load, the generated electrical stimulation can promote the migration of chondrocytes and the adsorption of extracellular proteins, and induce endogenous TGF-β through Piezo-induced Ca^2+^ influx, thereby improving cartilage formation and recovery.^157^ Piezoelectric materials have also been explored in tumor therapy through piezocatalytic and radiocatalytic strategies by enhancing ROS generation.^158^ However, these effects mainly rely on material-derived catalytic activity rather than confirmed Piezo-channel activation. Researchers considered the biodegradability of piezoelectric materials and developed a whitlockite (WH) nanomaterial, which has superior biodegradability compared to hydroxyapatite and tricalcium phosphate.^144^ When stimulated by low-intensity pulsed ultrasound, the WH nanomaterial can generate biological electrical signals resembling those in natural tissues, and it was found that it mainly promotes the proliferation and differentiation of mouse embryonic osteoblasts by increasing the expression of Piezo1 and TRPV4 pathways.^144^ Piezoelectric biomaterials represent an important bridge between external mechanical stimulation and biological electrical regulation. However, the generation of electrical signals by piezoelectric materials should not be directly equated with activation of Piezo channels. Future studies should clarify whether therapeutic effects originate from Piezo-dependent Ca^2+^ signaling, activation of other mechanosensitive channels, or broader electrochemical microenvironment regulation. The representative physical-field strategies associated with Piezo-mediated bioelectromagnetic modulation, including ultrasound-based stimulation, magnetic stimulation, piezoelectric biomaterial-based approaches, and mechanical loading, are summarized in Table 3. Furthermore, Fig. 4 provides a conceptual framework illustrating how different physical stimuli induce Piezo activation or Piezo-related bioelectrical cues, leading to Ca^2+^ influx/electrochemical signaling and subsequent tissue-specific responses.

**Table 3:** Representative physical-field strategies associated with Piezo-mediated bioelectromagnetic modulation.

Strategy	Physical stimulus	Piezo relevance	Mechanism (Piezo-mediated bioelectromagnetic modulation)	Representative applications	Reference
**Ultrasound-based strategies**	Low-intensity pulsed ultrasound (LIPUS); ultrasound-induced mechanical stress	High	Ultrasound mechanical stimulation or piezoelectric-material-generated electrical cues → activation of Piezo1/TRPV4 channels → Ca^2+^ influx → downstream signaling pathways (e.g., MAPK/ERK/BMP2 signaling) → tissue-specific physiological responses	Bone regeneration and osteogenic differentiation^144^; ultrasound-responsive piezoelectric nanomedicine and tumor therapy (Piezo-related or piezocatalytic strategies) 145, 146, 147, 148, 149	144, 145, 146, 147, 148, 149
**Magnetic stimulation-based strategies**	Static/dynamic magnetic field; magneto-mechanical force; magnetic nanostructure-induced mechanical deformation	High	Magnetic field → mechanical force generation or deformation of Piezo-associated cellular structures → Piezo1 channel gating → Ca^2+^ influx → activation of downstream signaling pathways (e.g., MAPK-related pathways) → physiological regulation	Remote neuronal modulation through Piezo-based magnetogenetic strategies^150^^,^^151^; Piezo1-mediated bone regeneration and matrix mineralization^155^; PiezoMagnetic nanoparticle-based multimodal therapy and tissue repair^156^	150,151,155,156
**Piezoelectric biomaterial-based strategies**	Mechanical loading (compression, shear stress, stretching); tissue-generated mechanical deformation	Moderate–High	Mechanical deformation of piezoelectric materials → generation of local electrical cues → Piezo-related mechanosensitive signaling modulation → Ca^2+^ influx/electrochemical responses → tissue regeneration or functional regulation	Bone regeneration and osteogenic stimulation through Piezo-related signaling^144^; cartilage repair using biodegradable piezoelectric scaffolds^157^; piezoelectric catalytic tumor therapy^158^	144,157,158
**Mechanical loading-based strategies**	Physiological mechanical loading; hydrostatic pressure; shear stress; substrate stiffness	High	Mechanical force → direct activation of Piezo1/Piezo2 channels → cation/Ca^2+^ influx → electrochemical signaling → regulation of cellular function and tissue homeostasis	Osteogenic differentiation and bone remodeling 18, 19, 20; vascular mechanotransduction and endothelial regulation 21, 22, 23; immune-cell activation and inflammatory regulation ^52^^,^^55^, ^56^, ^57^, ^58^, ^59^, ^60^, ^61^, ^62^; epithelial organ mechanosensation 30, 31, 32, 33, 34, 35, 36, 37, 38, 39, 40, 41, 42, 43, 44	18, 19, 20, 21, 22, 23,30, 31, 32, 33, 34, 35, 36, 37, 38, 39, 40, 41, 42, 43, 44,52,55, 56, 57, 58, 59, 60, 61, 62

**Note**: The level of “Piezo relevance” reflects the current evidence supporting direct Piezo-channel activation or Piezo-related bioelectrical/electrochemical modulation. Physical-field strategies with indirect or material-mediated effects are distinguished from those with experimentally validated Piezo-dependent mechanisms.

## 5 Conclusion

The Piezo channels, as the core molecule in living organisms that converts mechanical force stimulation into biological electrical signals, mark a milestone in the field of mechanotransduction. Compared with traditional pharmacological approaches targeting the Piezo channels, physical field-based interventions offer unique advantages in terms of non-invasiveness, high spatiotemporal resolution, and minimized off-target effects. Physical stimuli such as magnetic fields, ultrasound, and electrical signals can be precisely delivered to specific tissues or even deep brain regions with external control, enabling on-demand regulation of the Piezo activity without systemic drug exposure. This “physical therapy” paradigm not only circumvents the limitations of chemical drugs, such as poor bioavailability and unintended side effects, but also provides a platform for synergistic multi-modal treatments. Therefore, leveraging physical fields to modulate the Piezo channels represents a promising strategy for achieving precise, dynamic, and personalized interventions. This review, from a unique perspective of physical therapy, systematically elaborates on the exquisite “propeller-nano bowl” structure of the Piezo channels, the gating mechanism based on membrane tension and lever effect, and their extensive physiological functions in numerous systems ranging from bones, cardiovascular systems to nerves and immunity. Not only is it the natural “mechanical-electrical” signal converter in our body, but its dysfunction also directly leads to various diseases, including genetic disorders, osteoarthritis, and cancer. The biological electromagnetics technology provides interdisciplinary solutions for precisely regulating the Piezo: the biological electrical signals generated by barium titanate nanoparticles under ultrasound stimulation can promote osteogenic differentiation by activating the Piezo1, and its efficacy has been verified in a rabbit cartilage repair model. PiezoMagnetic nanoparticles integrate magnetic hyperthermia and ultrasound-triggered drug release, achieving dual effects of tumor ablation and bone regeneration in osteosarcoma treatment. By driving the mechanical gating of Piezo1 with a magnetic field, wireless regulation of neurons in deep brain regions has been successfully achieved, providing a new strategy for neurodegenerative diseases such as Parkinson's disease.

However, to fully translate the understanding of the Piezo channels into clinical applications, we still face a series of challenges. The millisecond kinetics of Piezo activation and the micrometer-level positioning requirements need to be combined with genetics and nano-mechanical devices to achieve dynamic monitoring. The Piezo1/2 in the compensatory mechanism of the cardiovascular system limits the specificity of drugs, and single-cell CRISPR screening is needed to analyze the tissue-specific functions. The Piezo1 regulates M1/M2 polarization in macrophages by sensing matrix hardness, and its synergy with the TGF-β pathway may become a new target for the treatment of inflammatory diseases. The mechanical-chemical coupling of Piezo1 with the YAP/TAZ pathway requires analysis of protein interaction networks to reveal the molecular mechanism.

**Fig. 5:**
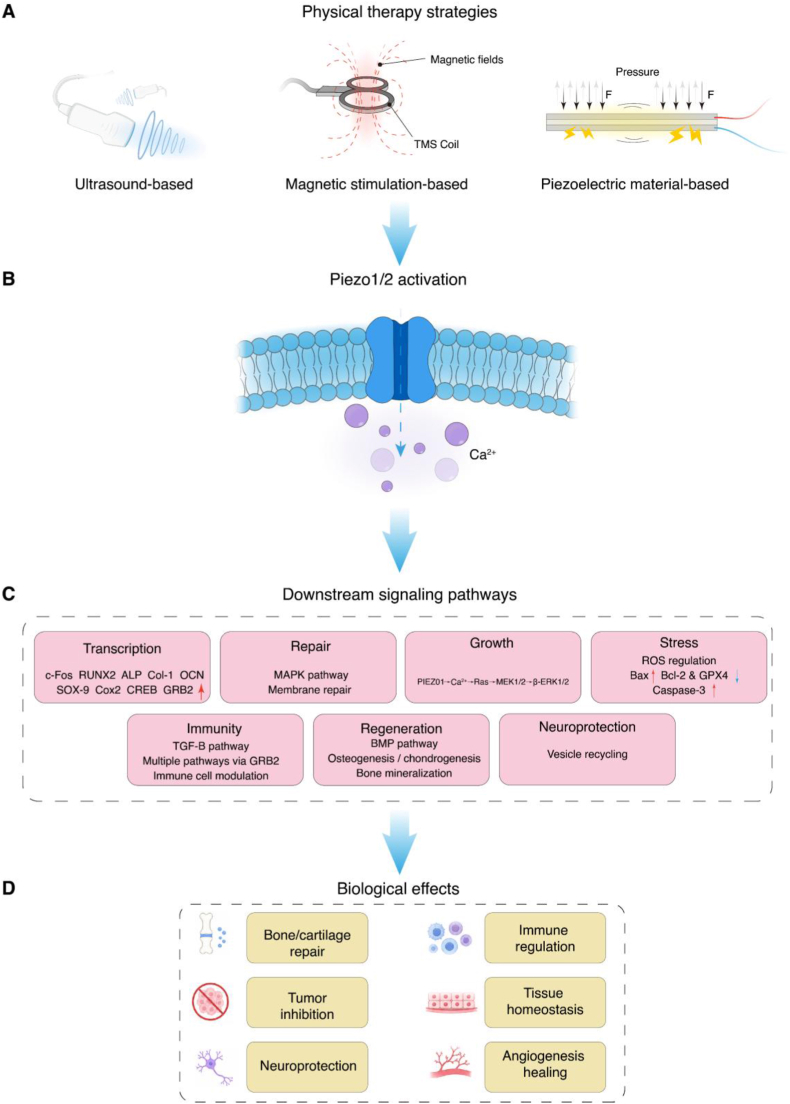
Mechanism diagram of physical therapy.

Developing tissue and cell-targeted delivery systems is the inevitable path to reduce systemic toxicity and improve efficacy. Meanwhile, we should actively promote the clinical application of piezoelectric biomaterials in regenerative medicine fields, including bone and cartilage regeneration, nerve repair, and wound healing. At present, many researchers have conducted studies on releasing hydrogel materials. Their excellent biological properties give them a natural advantage for use in the medical field, but they have not yet been applied in actual treatments 159, 160, 161, 162. The most promising direction lies in exploring strategies for coupling multiple physical fields (such as sound, magnetism, and electricity) to regulate the Piezo. In future experiments, we can use chemical substances such as the Piezo agonists Yoda1 and Jedi1/2, and the Piezo inhibitors GsMTx4, Dooku1, Aβ, ruthenium red, and Gd^3+^ as positive or negative controls to determine the effect of electromagnetic fields on the Piezo in cells ^47^^,^^163^, ^164^, ^165^, ^166^, ^167^, ^168^, ^169^. These interdisciplinary approaches will open a new window for us and will pave the way for a new “biomagnetic” treatment paradigm for treating pain, neurodegenerative diseases, cancer, and cardiovascular diseases. Fig. 5 is the mechanism diagram of physical therapy.

## Ethical approval

This study does not contain any studies with human or animal subjects performed by any of the authors.

## CRediT authorship contribution statement

**Bing Li:** Conceptualization, Data curation, Formal analysis, Writing – original draft. **Hui-Ming Kang:** Conceptualization, Methodology, Software, Writing – original draft. **Wei-Dong Pan:** Conceptualization, Funding acquisition, Resources, Supervision, Writing – review & editing. **Ye Li:** Conceptualization, Funding acquisition, Methodology, Resources, Supervision, Writing – review & editing.

## Declaration of competing interests

The authors declare that they have no known competing financial interests or personal relationships that could have appeared to influence the work reported in this paper.
